# Stroke in Saudi Arabia: epidemiology, prevention, clinical management, and emerging innovations—a narrative review

**DOI:** 10.3389/fmed.2026.1837691

**Published:** 2026-10-28

**Authors:** Abdullah A. Alshehri, Mohammad N. Alkhrayef, Rawan Alzaidi, Yahya M. K. Tawfik, Ahmad M. Aldossary, Musaad A. Altammami, Ibrahim A. Alradwan, Meteb M. Altaf, Fahad A. Almughem, Majed A. Majrashi, Mohammad N. Alomary, Abdullah O. Alshehry, Seham Almehmadi, Meshael Alturki, Nojoud AL Fayez, Ghalia H. Bakhsh, Mai M. Alzaydi, Mariam T. Tawfik, Fahad S. Al-Ajlan, Abdel Douiri, Adel Alhazzani, Essam A. Tawfik

**Affiliations:** 1 Advanced Diagnostics and Therapeutics Institute, Health Sector, King Abdulaziz City for Science and Technology, Riyadh, Saudi Arabia; 2 Disability Research Institute, Health Sector, King Abdulaziz City for Science and Technology, Riyadh, Saudi Arabia; 3 Department of Clinical Pharmacy, College of Pharmacy, King Saud University, Riyadh, Saudi Arabia; 4 Wellness and Preventative Medicine Institute, Health Sector, King Abdulaziz City for Science and Technology, Riyadh, Saudi Arabia; 5 Bioengineering Institute, Health Sector, King Abdulaziz City for Science and Technology, Riyadh, Saudi Arabia; 6 Pharmacy Services Administration, King Fahad Medical City, Riyadh, Saudi Arabia; 7 Emergency Department, King Faisal Specialist Hospital and Research Center, Riyadh, Saudi Arabia; 8 Neuroscience Center, King Faisal Specialist Hospital and Research Center, Riyadh, Saudi Arabia; 9 College of Medicine, Alfaisal University, Riyadh, Saudi Arabia; 10 School of Life Course and Population Sciences, Faculty of Life Sciences and Medicine, King’s College London, London, United Kingdom

**Keywords:** management, prevention, risk factors, Saudi Arabia, stroke

## Abstract

Stroke is a leading cause of disability and mortality worldwide and poses an increasing public health challenge in Saudi Arabia. This review summarizes national and regional data from Saudi Arabia on epidemiology, risk factors, clinical practices, and future directions of stroke management. The annual incidence of stroke is approximately 57 cases per 100,000 population, with higher rates among older adults and men. The overall number of stroke cases continues to increase, driven by population aging, demographic growth, and lifestyle-related risk factors. Hypertension, diabetes, obesity, dyslipidemia, and smoking are the principal modifiable risk factors, whereas age, sex, and genetics remain key non-modifiable contributors. Current challenges include weak primary prevention, limited public awareness, and insufficient emergency response systems. Although significant advances in acute stroke treatments, such as thrombolysis and thrombectomy, are widely available in tertiary centers, national access remains uneven, and disparities persist in secondary prevention and rehabilitation. Emerging innovations, including AI-based diagnostics, personalized treatment strategies, and integrated stroke care networks, are increasingly shaping clinical practice. Comprehensive national strategies are needed to strengthen prevention, optimize acute and long-term management, and inform policy development to reduce Saudi Arabia’s growing stroke burden.

## 1 Introduction

Stroke remains one of the leading challenges in global public health, representing a major cause of mortality, disability, and long-term socioeconomic burden (1). Stroke is an acute neurological deficit resulting from vascular occlusion or rupture. It is broadly classified into two types, ischemic and hemorrhagic, each with distinct mechanisms, clinical consequences, and management strategies. The World Health Organization (WHO) ICD-11 is further expanding it to include cerebral dysfunction with symptoms lasting less than 24 h or causing death (2). Ischemic stroke, which results from arterial obstruction by thrombus or embolus, accounts for approximately two-thirds of all cases worldwide, whereas hemorrhagic stroke, including both intracerebral and subarachnoid hemorrhage, contributes to a smaller but fatal share of the global burden (3). Despite advances in prevention and acute care, stroke continues to impose significant disability-adjusted life years (DALYs) and remains a major cause of death globally (4).

According to the Global Burden of Disease (GBD) 2021 estimates, stroke ranked as the third leading cause of death, resulting in over 7 million annual deaths and representing more than 10% of total global mortality (4, 5). It also ranked among the leading contributors to disability, accounting for approximately 160 million DALYs (6). While age-standardized incidence and mortality rates have declined in many parts of the world, the absolute number of cases continues to rise, driven mainly by aging populations, lifestyle changes, and increasing prevalence of vascular risk factors (7). It should be noted that more than 80% of the global stroke burden now falls upon low- and middle-income countries, where health systems often face limitations in stroke prevention, acute management, and rehabilitation services. Projections suggest that without intensified prevention efforts, annual stroke-related deaths could rise by approximately 50% by 2050, with consequent escalation in healthcare costs and socioeconomic strain (4, 8).

Saudi Arabia, like many rapidly developing nations, is undergoing a profound demographic and epidemiological transition (9). Increases in life expectancy, coupled with high prevalence of non-communicable diseases such as hypertension, diabetes, obesity, and dyslipidemia, have amplified the population's vulnerability to stroke (10). Recent estimates indicate that nearly three-quarters of the population is overweight, with over 40% classified as obese, key risk factors that substantially accelerate vascular disease (11).

Although comprehensive national surveillance systems are limited, available studies indicate a growing burden of stroke in the Kingdom. A systematic review and meta-analysis estimated the annual stroke incidence in Aseer Province at approximately 57 cases per 100,000 population, with regional findings indicating substantially higher rates among older adults and a marked increase in incidence among individuals aged over 80 years (12–14). Stroke-related mortality in Saudi Arabia is projected to nearly double by 2030, highlighting challenges such as insufficient public awareness, underdeveloped registries, and limited rehabilitation infrastructure (15).

The economic consequences of stroke in Saudi Arabia extend well beyond acute hospitalization, encompassing long-term rehabilitation needs, caregiver support, and loss of productivity (16). Notably, unlike the USA and EU countries, where stroke predominantly affects the elderly, a significant proportion of cases in Saudi Arabia occur among individuals of working age (17). This earlier onset amplifies both the social and economic consequences, with total estimated costs of approximately USD 9.2 billion in 2019 (18), underscoring the need for stronger prevention and intervention strategies (19). Despite the Kingdom's advanced healthcare vision under national transformation initiatives, health expenditure remains relatively modest compared to its income status, raising concerns about preparedness to tackle the growing burden of long-term conditions, including stroke (20).

This review provides a comprehensive narrative overview synthesizing current evidence on stroke incidence, prevalence, risk factors, and outcomes in Saudi Arabia and contextualizes these findings within regional and global patterns. It also examines healthcare system readiness, acute stroke care, rehabilitation capacity, and the current state of stroke surveillance, and highlights emerging priorities such as establishing comprehensive stroke registries, expanding telemedicine services, and adopting advanced rehabilitation technologies. It also highlights future research priorities in the context of Saudi Vision 2030 and its healthcare modernization objectives. The evidence was synthesized narratively rather than through a formal systematic review or meta-analytic process. Accordingly, the review provides a broad overview of the available literature and emerging trends while acknowledging variations in study design, geographical coverage, and the representativeness of the underlying evidence.

Relevant literature was identified through searches of major biomedical databases, including PubMed, Scopus, and Google Scholar. The search strategy combined terms related to stroke and cerebrovascular disease with terms addressing the Saudi Arabian context and relevant clinical and public-health domains. Search terms included “stroke,” “cerebrovascular disease,” “incidence,” “prevalence,” “risk factors,” “prevention,” “diagnosis,” “thrombolysis,” “thrombectomy,” “rehabilitation,” and “Saudi Arabia,” using appropriate combinations of keywords and Boolean operators. Eligible publications included original research articles, systematic reviews, meta-analyses, clinical guidelines, and relevant national or institutional reports addressing stroke in Saudi Arabia. Priority was given to recent publications, Saudi-based studies, and evidence directly relevant to the epidemiology, prevention, diagnosis, treatment, rehabilitation, and healthcare delivery of stroke. The review also considered international studies and clinical guidelines when they provided essential clinical context or supported the interpretation of Saudi findings. Publications unrelated to the scope of the review, duplicate records, and sources lacking sufficient relevant information were excluded. The authors assessed the identified literature for relevance to the review objectives, focusing on geographical setting, study population, study design, and the applicability of reported findings to the Saudi healthcare context.

## 2 Stroke types and epidemiology in Saudi Arabia

### 2.1 Incidence and prevalence

Stroke has emerged as a significant healthcare challenge in Saudi Arabia, driven by population aging, rapid urbanization, and the growing prevalence of chronic diseases (16, 21). Over the past decades, the incidence of stroke has risen significantly, mainly due to the high burden of non-communicable conditions such as hypertension, diabetes, and obesity (13).

Available Saudi studies suggest a substantial stroke burden; however, the lack of comprehensive, longitudinal, population-level surveillance limits the ability to quantify national temporal trends reliably. Despite differences in methods and sampling limitations, estimates in Saudi Arabia suggest collective annual rates of 57 per 100,000 people (14, 22). Without targeted prevention, stroke incidence is projected to continue rising in the coming years, driven by demographic aging and the persistent prevalence of modifiable risk factors (16). National data on stroke subtypes in Saudi Arabia show that ischemic stroke is reported as the most common subtype, accounting for about two-thirds to three-quarters of cases; intracerebral hemorrhage accounts for most of the remaining burden, whereas subarachnoid hemorrhage remains rare, typically reported in only 2%–4% of cases (13, 14, 22). These proportions vary by study setting and population samples. Clinical and tertiary centers report a higher proportion of hemorrhagic strokes in young adults, which may be attributed to genetic factors and differing exposure patterns. However, genetic factors may contribute to stroke susceptibility in selected Saudi populations, but their population-level contribution relative to established vascular risk factors requires further investigation (23).

In Saudi Arabia, stroke primarily affects men, representing approximately 60% of reported cases demographically (13). Despite increasing awareness and some shifts in health behaviors, the higher incidence among men may be explained by higher rates of smoking and metabolic disorders (24). Moreover, there are increasing cases of individuals who get early-onset stroke under 60 years old (23), raising growing concerns that need attention. These observations are consistent with national data showing a significant number of adults experiencing obesity and diabetes, which consequently increases stroke risk (13, 24).

In a registry-based analysis, ischemic stroke had a prevalence of 4.42% among adults with diabetes, with a higher occurrence among those with hypertension or longer disease duration (24). The data reported are likely underrepresentation of the actual burden of stroke, as rural populations are often not considered due to limitations in surveillance systems and barriers to access in these regions (16, 21). Other factors contributing to underreporting and treatment delays include low awareness of stroke symptoms and insufficient availability of stroke centers in smaller cities (25), leading to delays in early intervention and distortion of national data. There has been a call for developing a centralized stroke registry to address existing gaps and enhance the accuracy of incidence tracking, precise characterization of strokes, and improved evaluation of the national health system's performance (14).

Ultimately, the reported evidence highlights the growing burden of stroke in Saudi Arabia, driven by its demographic profile, dominant risk factors, and gaps in the healthcare system. Ischemic stroke is at its peak, but a shifting trend toward younger adults and regional differences requires urgent attention and a need to enhance prevention, registry development, and increased accessibility to enable better mitigation and reduce the long-term impact of stroke on individuals and the health system.

### 2.2 Temporal trends

There is a clear and concerning divergence in stroke incidence between age groups in high-income countries throughout the last four decades. The incidence of stroke in older adults (typically defined as those 55 and older) has been significantly decreasing by 42% (26). However, this progress is not mirrored in younger populations (age <55 years); stroke incidence in those under 55 is actively increasing in the US and some other countries (26).

A comprehensive systematic review and meta-analysis led by Rothwell et al. revealed a significant age-specific divergence in stroke incidence trends. While stroke rates have generally been falling in older populations, this decline is not being seen in adults younger than 55 (26). The analysis of 50 studies across 20 countries has confirmed that almost all studies showed a less favorable trend in stroke incidence, consistent for both ischemic and hemorrhagic strokes at younger ages compared to older populations (26, 27). The study showed that this shift is a significant public health concern, highlighting that the progress made in preventing vascular events is not extending to younger ages. The surprising increase in stroke among younger generations, despite a general fall in incidence at older ages, is similar to trends seen in other diseases like colorectal cancer. Both conditions share risk factors such as obesity, poor diet, and lack of exercise, which are becoming more prevalent in younger generations (26, 28).

Traditional vascular risk factors like high blood pressure and cholesterol were poorly managed in young stroke patients, particularly in women and those with mental health conditions; however, they do not fully account for the rising incidence of stroke in this age demographic. This is partly because current treatment guidelines, based on risk prediction models, often lead to under-treatment in younger generations, whose overall risk is considered low (29). While heart attacks are becoming less common in the young generation, stroke incidence is increasing in this age group. This suggests that the underlying risk factors causing the increase in stroke incidence in the young population remain unknown (27).

Emerging vascular risks like air pollution and long working hours seem to affect stroke risk differently depending on age. Long work hours, for example, are more closely associated with stroke than with myocardial infarction (30). Although the increase in stroke incidence seems to be similar for both genders, which suggests that factors for age-specific conditions like pregnancy or oral contraceptive use are possibly not the leading cause, the text acknowledges that trends in women are more varied, probably because of changes in hormone replacement therapy use over time (31–33).

In Saudi Arabia, meta-analytic evidence estimates the overall annual stroke incidence at roughly 57 per 100,000 for first-ever hospitalized stroke cases. Although stroke incidence remains low among adults younger than 40 years, an apparent age-dependent rise persists, highlighting age as a major risk factor (12–14). However, unlike many high-income countries with established longitudinal stroke surveillance systems, Saudi Arabia lacks national, population-level trend data, limiting the ability to determine whether the global deviation in age-specific stroke incidence is also occurring locally.

Nevertheless, demographic and lifestyle trends in Saudi Arabia suggest similar shifts may occur in the coming decades. The Saudi population is relatively young, and the rapid rise in metabolic syndrome, including obesity, diabetes, and physical inactivity, combined with increasing urbanization, creates conditions that may elevate stroke risk among younger adults (34). These factors highlight the need for a national stroke registry or surveillance program to track age-specific incidence, detect early shifts in younger populations, and target prevention strategies aligned with Vision 2030's focus on non-communicable disease prevention.

### 2.3 Mortality and burden of stroke in Saudi Arabia

Stroke is a leading health challenge in Saudi Arabia, contributing substantially to mortality, long-term disability, and healthcare demand. National and regional data show that the one-year mortality rate following stroke ranges between 14% and 27%, which is higher than reported in some neighboring Gulf countries. The reasons for this difference are likely multifactorial, including variations in acute stroke service availability, pre-hospital systems, and early rehabilitation access (35, 36).

In terms of public health impact, stroke is the second leading cause of death in the Kingdom, surpassed only by ischemic heart disease (4). This reflects both the growing prevalence of non-communicable diseases and the increasing proportion of older adults in the Saudi population. As survival rates for other cardiovascular diseases improve, stroke is emerging as a major cause of life years lost and a key driver of DALYs nationally (5).

The consequences of stroke extend far beyond the acute event. A significant proportion of survivors live with long-term disability, including weakness, speech impairment, cognitive decline, and loss of independence. Many require ongoing assistance for activities of daily living, either from family caregivers, who form the cornerstone of post-stroke support in Saudi society, or from institutional care providers (37). This dependency imposes both emotional and financial strain, often persisting for years after the initial event.

From a social perspective, the burden of stroke is amplified by reduced workforce participation, extended rehabilitation needs, and readmissions for complications such as pneumonia, deep vein thrombosis, and recurrent strokes (38, 39). The healthcare system faces a growing demand for specialized stroke units, long-term rehabilitation services, and community-based support programs to meet these needs (40).

In summary, stroke in Saudi Arabia is characterized by high early mortality and significant long-term disability, placing it among the foremost contributors to death and disability in the Kingdom. Reducing this burden will require a national focus on prevention, rapid acute care, and stronger rehabilitation and community support pathways. Despite the increasing availability of epidemiological data, the absence of a unified stroke surveillance system limits the accuracy of incidence estimates. It hinders longitudinal monitoring of regional disparities, temporal trends, and outcomes. Most currently available data are derived from single-center or regional studies, which may not fully represent the national burden of stroke across diverse populations in Saudi Arabia.

### 2.4 Stroke types and classification

Stroke is defined by the abrupt onset of focal neurological deficits due to vascular pathology (41). Clinically, strokes are classified into two principal categories, as shown in Figure 1: ischemic stroke and hemorrhagic stroke, the latter comprising intracerebral hemorrhage and subarachnoid hemorrhage (42). Although transient ischemic attack is not formally considered a stroke under the WHO and the Saudi Stroke Society guidelines, it is conventionally included in the broader spectrum of cerebrovascular disease because of its close clinical overlap and prognostic importance (17). While distinct from stroke, transient ischemic attacks act as critical warning events, often preceding major strokes, and thus remain central to discussions of stroke classification, acute triage, and secondary prevention (42, 43).

**Figure 1:**
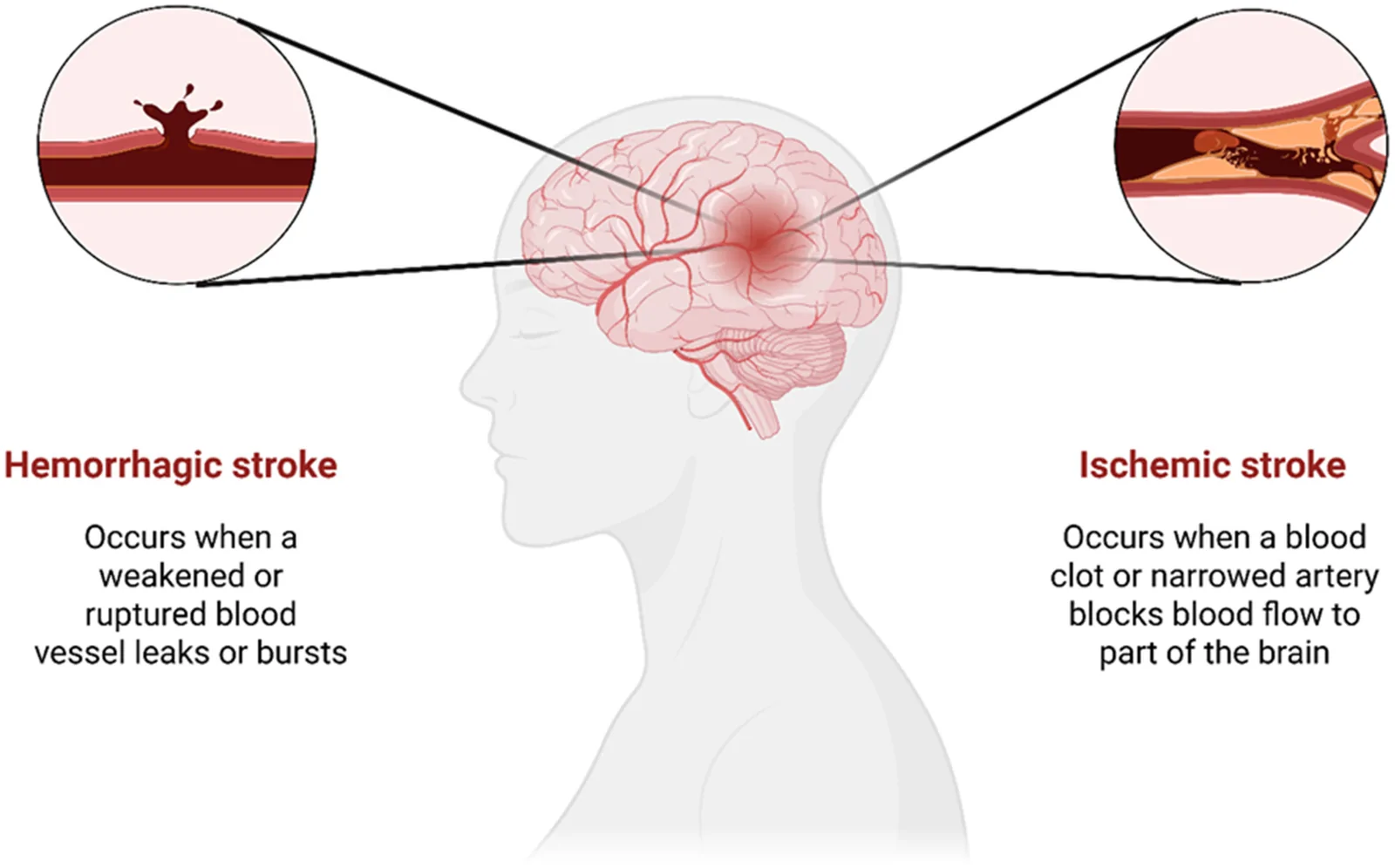
Illustration of the two main types of stroke and their effects on cerebral blood flow. Ischemic stroke (right) is illustrated as a narrowed or blocked artery caused by a blood clot, restricting blood flow to part of the brain, whereas hemorrhagic stroke (left) is depicted as a weakened blood vessel that ruptures and leaks blood into the surrounding brain tissue. Created with BioRender.com.

Ischemic stroke is the predominant form, accounting for approximately 80% of cases both worldwide and in Saudi Arabia (44). It arises from arterial occlusion, which reduces cerebral blood flow. When flow declines below ∼15–20 mL/100 g/min, irreversible infarction occurs, whereas tissue perfused at ∼20–40 mL/100 g/min constitutes the ischemic penumbra, a functionally impaired but structurally viable zone that is potentially salvageable with timely reperfusion (43, 45). This concept supports modern acute therapies, including intravenous thrombolysis and endovascular thrombectomy.

Etiologically, the Trial of Org 10172 in Acute Stroke Treatment (TOAST) classification, first developed in 1993 during a randomized controlled trial of the antithrombotic agent Org 10172, has become the most widely used etiologic framework for ischemic stroke. It distinguishes large-artery atherosclerosis (20%–25%), cardioembolism (20%–30%, often due to atrial fibrillation), small-vessel occlusion (20%–25%), other determined causes (5%–10%), and cryptogenic strokes (20%–40%), the latter referring to ischemic strokes of undetermined origin despite comprehensive diagnostic evaluation (46). Many cryptogenic strokes are now thought to arise from covert embolic mechanisms, including occult atrial fibrillation or a patent foramen ovale (PFO), a congenital cardiac defect characterized by a persistent opening between the right and left atria that allows paradoxical emboli to reach the cerebral circulation (47, 48).

The Oxfordshire Community Stroke Project (OCSP) provides a complementary topographic framework, categorizing infarcts into anterior circulation (total or partial), lacunar, and posterior circulation syndromes (49). Contemporary imaging-based models add further precision by incorporating large-vessel occlusion status, perfusion-diffusion mismatch, and collateral integrity, which directly inform eligibility for reperfusion and neuroprotective interventions (50). In Saudi cohorts, large-artery and small-vessel occlusions predominate, whereas cardioembolic and cryptogenic subtypes are under-represented, reflecting limited use of long-term cardiac monitoring. Importantly, the median age of ischemic stroke is ∼59 years, nearly a decade younger than in Western populations, driven by high burdens of diabetes (58%), hypertension (72%), and obesity (>35%) (51).

In contrast to ischemic stroke, which predominates globally, hemorrhagic stroke is less common but carries a disproportionate burden of mortality. Hemorrhagic strokes comprise ∼15% of cases globally and 11%–15% in Saudi Arabia, but are associated with disproportionately high morbidity and mortality (52). The intracerebral hemorrhage results from rupture of small penetrating arteries, most often due to chronic hypertension or, in elderly populations, cerebral amyloid angiopathy. Hematoma volume (>30 mL) and location (lobar vs. deep) are the strongest prognostic factors, with global 30-day mortality rates >40% (53). In Saudi Arabia, ∼70% of intracerebral hemorrhage cases are linked to uncontrolled hypertension, with mean presenting systolic blood pressures >180 mmHg and 30-day case fatality rates approaching 28% (54). The subarachnoid hemorrhage accounts for 2%–3% of Saudi strokes and is most frequently aneurysmal. Rupture results in diffuse meningeal bleeding, abrupt rises in intracranial pressure, and secondary global ischemia. Complications include vasospasm, delayed cerebral ischemia (in up to 30% of survivors), hydrocephalus, and rebleeding (55). Saudi data mirror global observations in which ∼85% of subarachnoid hemorrhage cases are aneurysmal, and ∼60% occur in women (56).

Transient ischemic attacks are brief episodes of focal neurological dysfunction caused by transient cerebral ischemia without infarction. A tissue-based definition, emphasizing the absence of infarction on diffusion-weighted Magnetic resonance imaging (MRI), is now favored over the traditional time-based threshold of <24 h (14). Up to 15%–30% of ischemic strokes are preceded by a transient ischemic attack, and the early risk of stroke after a transient ischemic attack is ∼5% within 48 h, highlighting the importance of rapid recognition and secondary prevention (23). In Saudi Arabia, transient ischemic attacks represent ∼3%–5% of cerebrovascular presentations, though underdiagnosis is likely, as only 27% of patients present within 3 h of symptom onset (57).

Epidemiological data from Saudi Arabia mirror global patterns but also reveal essential national distinctions. Nearly one-quarter of strokes occur in patients <50 years, disproportionately affecting men (14). Gender differences are evident, with hemorrhagic strokes more frequent in males, while older women more often present with cardioembolic ischemic stroke (14). Systemic barriers, including delayed hospital arrival, limited access to stroke-ready centers, and low thrombolysis rates (<10%), remain critical challenges, particularly in rural regions (58, 59). National initiatives, however, are actively addressing these gaps: expanding specialized stroke hospitals, rolling out hyperacute protocols (IV thrombolysis and thrombectomy), and developing the Saudi Stroke Registry are expected to improve recognition, access equity, and overall outcomes (60–62). Taken together, stroke classification integrates type (ischemic stroke, hemorrhagic stroke, and transient ischemic attacks), etiology (TOAST), vascular territory and syndrome (OCSP), pathophysiology and imaging-based biomarkers (penumbra, large-vessel status, perfusion mismatch), and functional severity (e.g., National Institutes of Health Stroke Scale [NIHSS], modified Rankin Scale [mRS]) (43, 63, 64). This multidimensional framework bridges clinical taxonomy with neurovascular unit biology, enabling precision in diagnosis, therapeutic decision-making, and secondary prevention strategies.

### 2.5 Regional and global context of stroke burden and care

Saudi Arabia's stroke burden should be interpreted within the broader epidemiological transition occurring across the Gulf Cooperation Council (GCC), Middle Eastern, and North African (MENA) countries, where population growth, aging, and cardiometabolic risk factors continue to drive rising stroke burden (65, 66). In Saudi Arabia, the annual stroke incidence is about 57 per 100,000, based on national and regional data (14, 66). In the GCC countries, there is substantial heterogeneity, with the United Arab Emirates (UAE) reporting the highest stroke incidence in the GCC at 106 per 100,000, and Bahrain the lowest at 47 per 100,000 (14, 66). Between 1990 and 2021, the absolute number of annual stroke cases in the MENA region rose by 101.2% to over 614,800, driven primarily by population growth, aging, and persistent modifiable risks including high systolic blood pressure, dietary risks, air pollution, diabetes, and dyslipidemia (14, 66–68). In contrast, high-income developed countries exhibited a markedly divergent public health trajectory, marked by a historical 42% reduction in overall stroke incidence and significant declines in age-standardized mortality rates (Table 1) (69). Compared to many Western high-income countries, stroke patients in MENA tend to be younger and have higher rates of diabetes and metabolic disease, increasing long-term socioeconomic burden (70).

**Table 1:** Overview of stroke burden, risk factors, and care indicators in Saudi Arabia, GCC/MENA, and developed countries.

Domain	Saudi Arabia	GCC/Arab/MENA	Global/developed-country
Annual stroke incidence	∼57 per 100,000 (56)	UAE 106, Kuwait 71, Oman 69, Qatar 48, Bahrain 47 per 100,000 (66)	11.9 million incident strokes (4, 71).
Stroke prevalence and Age-Standardized Prevalence Rate (ASPR)	965 cases per 100,000 people based on estimates and modeling, a national registry is recommended (72)	MENA prevalent cases increased from 2.58 million to 5.57 million between 1990 and 2021; ASPR declined from 1,210 to 1,074 per 100,000 (65)	93.8 million prevalent stroke cases (73)
Stroke deaths	Saudi stroke death rate: 36 per 100,000, decreased from 49.86 per 100,000 in 1990 (66)	GCC 2021 death rates per 100,000: Bahrain 23, Oman 21, Kuwait 16, UAE 12, Qatar 8. MENA death declined from 170 to 101 per 100,000 from 1990 to 2021 (65, 66)	7.3 million stroke deaths, accounting for ∼10.7% of all global deaths (73)
Disability-Adjusted Life Year (DALY) burden	Highest GCC rate in 2021: 1,164 per 100,000 (14)	MENA stroke DALYs increased in absolute number, but age-standardized DALY rates declined (66, 70)	160.5 million DALYs due to stroke (73)
Ischemic stroke proportion	Ischemic stroke proportions of 65%–76% (14)	Ischemic stroke remains the main subtype in Arab and MENA cohorts, 79%–85% (70)	Dominant global subtype 65%–80%, distribution can vary by region & data source (73)
Age/clinical profile	Younger clinical profile with a median ischemic stroke age of ∼57–63 years (74)	MENA patients 59–65 years (70, 75)	Stroke burden concentrated among older adults aged 66–73 years (73)
Acute reperfusion standards	Saudi Stroke Standards: reperfusion assessment within 24 h; IV thrombolysis within 4.5 h; mechanical thrombectomy within 24 h (76)	Reperfusion assessment within 24 h; IV thrombolysis within 4.5 h; mechanical thrombectomy 6–24 h, resource-capable centers may vary (65)	UK/USA Stroke Standards: Reperfusion assessment within 24 h; IV thrombolysis within 4.5 h (up to 24 h via perfusion); mechanical thrombectomy within 24 h (73)
Registry/audit infrastructure	Recommends a stroke registry to monitor incidence and improve stroke services (14)	MENA registry maturity remains variable in comprehensive population-level surveillance (65)	37 national hospital-based stroke registries from 31 countries; most were in high-income countries (67)

GCC, Gulf Cooperation Council; h, hour; IV, intravenous; MENA, Middle Eastern and North African; UAE, United Arab Emirates; UK, United Kingdom; USA, United States of America.

These epidemiological differences are mirrored by disparities in acute stroke care and long-term prevention systems. Saudi Arabia has expanded tertiary stroke care, including intravenous thrombolysis, mechanical thrombectomy, advanced neuroimaging, stroke units, and emerging telestroke services (60). However, access remains uneven, with delayed presentation, low awareness, and fragmented pathways hindering timely reperfusion (72). Many developed healthcare systems use stroke registries, quality indicators, referral pathways, and prevention programs to improve outcomes (67). Saudi Vision 2030 offers a key framework for healthcare modernization, but priorities remain, including strengthening stroke surveillance, improving regional access, expanding prevention efforts for modifiable risk factors, and developing integrated rehabilitation and secondary prevention pathways (17, 72).

## 3 Risk factors and underlying causes

### 3.1 Risk factors

Stroke is influenced by both modifiable and non-modifiable factors, as shown in Figure 2, and, according to GBD 2025, over 80% of strokes worldwide are attributable to preventable exposures, highlighting the critical role of prevention (4, 77). Figure 2 highlights the overlap between modifiable vascular risk factors and regional epidemiological variation, emphasizing the potential value of region-specific prevention strategies and targeted public health interventions. While hypertension, diabetes, obesity, smoking, and atrial fibrillation (AF) dominate globally, Saudi Arabia faces additional challenges, including high consanguinity, widespread shisha (waterpipe or hookah) use, extreme climate conditions, and changing dietary habits (78).

**Figure 2:**
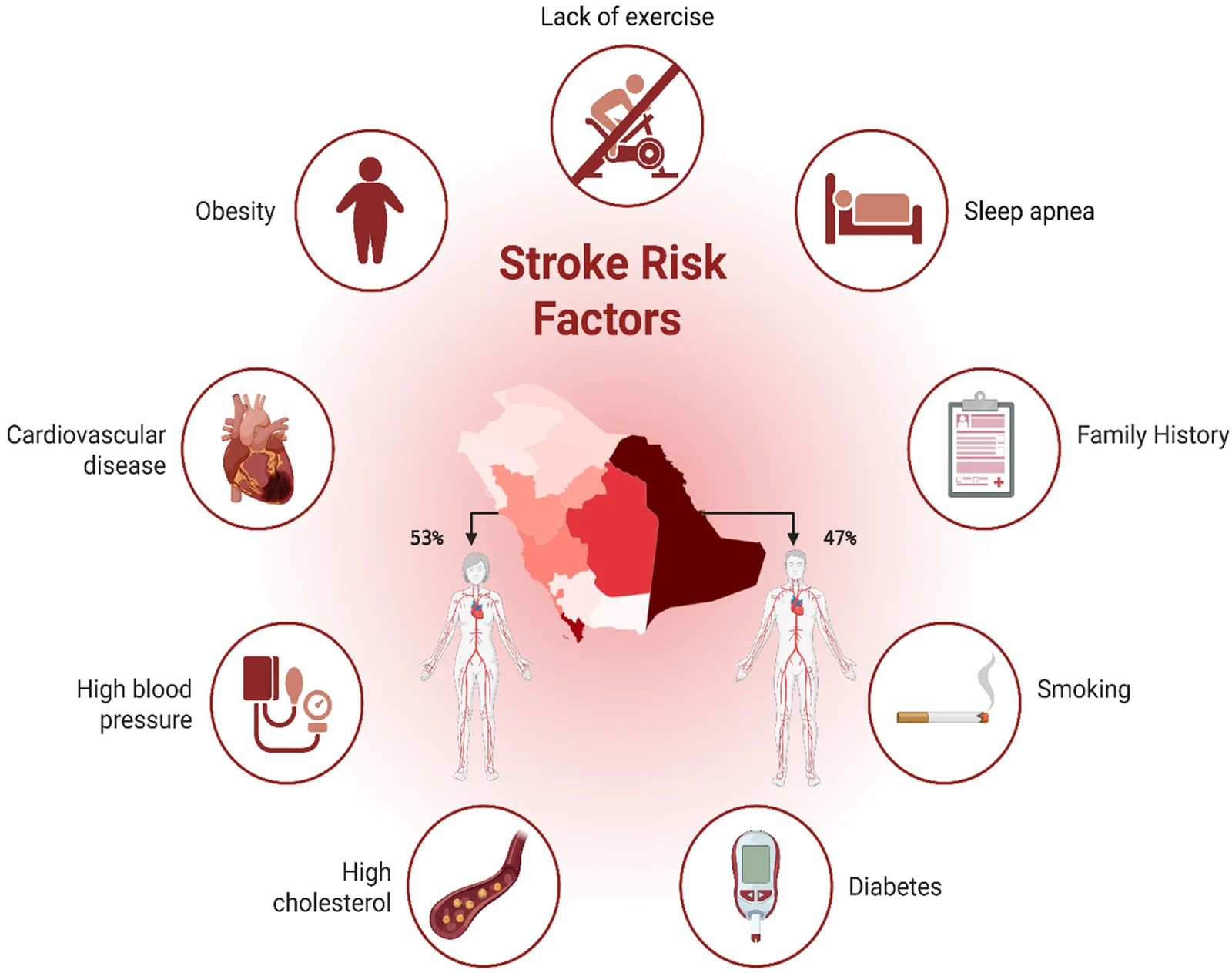
Stroke risk factors, gender-based prevalence, and regional epidemiology in Saudi Arabia, with higher rates observed in the Eastern and Jizan regions. The illustration presents the major modifiable and non-modifiable risk factors associated with stroke, including hypertension, diabetes, high cholesterol, smoking, cardiovascular disease, obesity, physical inactivity, sleep apnea, and family history, emphasizing the burden and population-level impact of stroke risk factors. Created with BioRender.com.

#### 3.1.1 Modifiable risk factors

Modifiable traditional factors remain the most impactful globally and locally. Hypertension is the leading risk, with elevated systolic blood pressure and cumulative burden linked to both ischemic and hemorrhagic stroke (79, 80). In Saudi cohorts, hypertension is the most common comorbidity, affecting over half of stroke patients, and the proportion of strokes caused by hypertension is probably even higher than the one-third reported in high-income countries (44, 81). Diabetes increases stroke risk by 30% to 40%, and the presence of diabetic retinopathy is linked to nearly double the risk; Saudi data show that higher glycated hemoglobin (HbA1c) levels are particularly associated with ischemic stroke (82, 83). Dyslipidemia and obesity add further risk, with adiposity consistently linked to the first stroke (84). Surveys in Saudi Arabia show that many stroke survivors struggle with obesity, physical inactivity, and dyslipidemia, underscoring the need for more effective prevention strategies (14). Smoking increases the risk of stroke, and shisha use could pose an even greater risk. A 2024 study reported that people who use shisha had almost three times the odds of stroke compared with those who do not (85). With rising popularity among Saudi youth, shisha use has become a pressing prevention target. AF is also a major driver, raising stroke risk more than twofold and leaving a residual annual risk of 1.5% to 2.5% even in anticoagulated patients (86, 87). In Saudi Arabia, AF is often underdiagnosed and undertreated, highlighting gaps in screening and anticoagulation use (88).

#### 3.1.2 Non-modifiable risk factors

Non-modifiable factors include age, sex, family history, and genetic predisposition. The risk of stroke increases steeply with age, making older age the strongest predictor for both ischemic and hemorrhagic types. GBD data show that men tend to have strokes at younger ages, while women are more often affected later in life and with greater severity (4, 77). Women with AF are at even higher risk of thromboembolic events than men (89); and genetic factors further increase overall vulnerability. A positive family history is also recognized as an independent risk factor, reflecting shared genetic and environmental influences (90). In Saudi Arabia, consanguinity rates exceed 50%, increasing the contribution of recessive variants (91). Whole-exome sequencing in Saudi ischemic stroke cohorts has identified rare variants such as COL4A1/2, ADA2, and Fabry disease–related genes, which explain some cases of young or cryptogenic stroke (92). The latter affects one-third of ischemic strokes and has a substantial clinical burden. Although the attributable fraction of genetic risk is modest compared with hypertension or diabetes, it remains a distinctive feature of stroke epidemiology in the Kingdom.

### 3.2 Prevention strategies

Preventing stroke not only reduces incidence but also improves functional outcomes, lessens health-system costs, and enhances quality of life. Stroke prevention is most effectively organized around a three-level hierarchy: primordial, primary, and secondary prevention, which reflects the temporal distance from disease onset and the type of intervention required. This framework, endorsed by the American Heart Association/American Stroke Association (AHA/ASA) guideline (93), the World Stroke Organization (WSO)- Lancet Neurology Commission (94), and national strategies in different countries, including Saudi Arabia (17), provides a common language for clinicians, public-health officials, and policymakers to coordinate actions across the health-care continuum.

Primordial prevention targets the social, economic, environmental, and policy determinants that underlie the classic vascular risk factors (hypertension, dyslipidemia, diabetes, smoking, obesity, physical inactivity). At this stage, the goal is not to treat an existing abnormality, but to prevent its emergence (17). World Stroke Organization Commission defines primordial measures as actions aimed at enhancing social and economic conditions, promoting healthier urban environments and housing, ensuring universal health coverage, making nutritious food affordable, and lowering exposure to air pollution, tobacco, excess salt, sugar, and trans fats (8).

Evidence from the INTERSTROKE study identified ten modifiable risk factors that together account for roughly 90% of stroke risk worldwide, demonstrating that targeting the upstream drivers of these factors can produce a dramatic shift in the population-level risk distribution (95). In practice, this translates into a suite of policy and environmental actions. Fiscal measures such as taxes on tobacco, sugary beverages, salt, and trans-fat not only reduce consumption but also generate revenue that can be reinvested in health services (8). Urban design interventions that create walkable neighborhoods, cycling lanes, and green spaces encourage regular physical activity while simultaneously lowering exposure to air pollution (8). Regulatory approaches that mandate reductions in sodium content in processed foods and restrict the advertising of unhealthy items further shape the food environment toward healthier choices (8). In addition, education and early-life programs, such as school-based nutrition curricula and community-wide health promotion campaigns, instill healthy habits from childhood, laying a durable foundation for lifelong stroke prevention (17).

In Saudi Arabia, the Vision 2030 health-promotion agenda incorporates these elements through the Saudi Green Initiative, taxation on sugary beverages, and the development of “healthy cities” that provide accessible sports facilities and clean air (17). The overarching premise is that when the environment makes the healthy choice the easy choice, the incidence of hypertension, obesity, and dyslipidemia declines, thereby reducing the pool of individuals who ever require primary or secondary prevention. Moreover, the Health in All Policies (HiAP) framework adopted in Saudi Arabia provides an essential upstream policy foundation for stroke prevention, despite stroke not being explicitly addressed in the framework. By recognizing that approximately 80% of population health determinants lie outside the healthcare sector, HiAP emphasizes multisectoral interventions targeting education, urban and rural environments, food systems, income and living conditions, and social contexts-factors that are strongly linked to major modifiable stroke risk factors such as hypertension, obesity, physical inactivity, and unhealthy diets (96).

Primary prevention focuses on people who already possess one or more modifiable risk factors but have not yet experienced a cerebrovascular event. The 2024 AHA/ASA guideline recommends systematic screening for blood pressure, fasting glucose, lipid profile, and AF at every primary-care encounter, with treatment thresholds that often require multiple antihypertensive agents to achieve targets < 130/80 mmHg (93). Lifestyle modification remains the cornerstone: adherence to a Mediterranean-style diet, ≥ 150 min/week of moderate-intensity aerobic activity, smoking cessation, and moderation of alcohol intake have been shown to reduce first-stroke risk by up to 80% when applied collectively (97). In addition, precision-medicine approaches, including the development and application of population-specific polygenic risk scores tailored to the Saudi population, may further enhance primary stroke prevention by enabling the early identification of genetically high-risk individuals who could benefit from earlier, targeted, or more intensive risk-factor management.

Pharmacologic tools complement behavioral change. Statins are indicated for anyone with a 10-year ASCVD risk ≥7.5% (98). In settings where polypharmacy hampers adherence, a fixed-dose combination “polypill” (low-dose antihypertensive and statin) offers a pragmatic, cost-effective solution, especially in low- and middle-income countries (8). Primary prevention also embraces sex-specific and disease-specific nuances. The AHA/ASA guideline adds recommendations for women with migraine with aura, patients with sickle-cell disease, and individuals with high-risk pregnancy conditions, recognizing that these groups have distinct pathophysiologic pathways to stroke (93).

Secondary prevention targets stroke survivors, a group whose risk of a recurrent event is 6 to 15 times higher than that of the general population (94, 99). The primary objectives are (i) identifying the underlying etiology, such as large-artery atherosclerosis, cardioembolism, and small-vessel disease, and (ii) implementing intensive evidence-based therapy to mitigate the cause (8). Antithrombotic treatment forms the cornerstone of medical management: patients with non-cardioembolic stroke receive antiplatelet agents, those with AF are prescribed oral anticoagulants, and a subset of high-risk minor strokes may benefit from short-term dual antiplatelet therapy (100). Blood pressure control is equally critical, with current guidelines recommending a target of less than 130/80 mmHg, a level that often requires combination antihypertensive regimens (93). Lipid-lowering therapy with high-intensity statins is advised for essentially all stroke survivors unless contraindicated (17). Emerging precision-medicine strategies, including pharmacogenetic testing, offer significant opportunities to optimize secondary stroke prevention. Genotype-guided selection and dosing of antiplatelet and anticoagulant therapies, such as identifying CYP2C19 loss-of-function variants associated with reduced clopidogrel efficacy, enable more precise alignment of treatment with individual metabolic profiles, thereby improving therapeutic effectiveness and reducing adverse drug reactions (101). In practice, suboptimal medication adherence and gaps in follow-up continue to be significant barriers, especially in low- and middle-income settings (8). For patients who have symptomatic carotid artery stenosis exceeding 50%, prompt revascularization, either carotid endarterectomy or stenting, significantly reduces the likelihood of recurrence (102). More details will follow in the upcoming sections.

Medical therapy alone, however, is insufficient without a structured system of post-stroke care. Comprehensive rehabilitation programs, early-supported discharge pathways, and the use of “stroke passports” that compile each patient's risk-factor profile, medication list, and follow-up schedule are essential for maintaining long-term control (102). To operationalize these goals, the Irish National Stroke Strategy mandates rapid-access transient ischemic attacks clinics that evaluate patients within 24 h and provide multidisciplinary follow-up to ensure that secondary-prevention targets are achieved (102). Saudi Stroke Pathway Standards state that without an integrated post-stroke care system that includes acute stroke units, multidisciplinary rehabilitation, and coordinated follow-up, pharmacological treatment alone cannot achieve optimal secondary prevention outcomes (100).

Although presented as distinct stages, the three pillars are interdependent. Robust primordial policies, such as reduced sodium in processed foods, lower the prevalence of hypertension, thereby shrinking the pool of individuals who require primary-prevention treatment (103). Conversely, effective primary-prevention programs that achieve high adherence to antihypertensive and lipid-lowering therapy reduce the number of patients who progress to stroke and subsequently need secondary-prevention services (93). Finally, secondary prevention programs that achieve high medication adherence and lifestyle modification can feed back into primordial goals by demonstrating the health-economic benefits of upstream interventions, thus strengthening political will for policy change (104). The three pillars of stroke prevention, primordial, primary, and secondary, constitute the full spectrum of individual-level actions that clinicians should deliver at the point of care.

Public-health campaigns and education are essential for raising awareness of stroke symptoms (FAST) and risk factors. In Ireland, the National Stroke Strategy 2022–2027 mandates at least two professional and two public education programs per year, integrated across the stroke-care pathway (105). Texas's “Public Health Strategies for Addressing Heart Disease and Stroke” similarly set targets to increase symptom-awareness from 15.5% to 16.3% (106). Recent survey data showed that Saudi adults have only moderate awareness of stroke risk factors and warning signs (around 60% overall), highlighting the urgency of expanding public-education efforts to improve early recognition and prevention (107). This aligns with the Saudi Stroke Pathway Standards, which stress that public and community-awareness campaigns are essential for early presentation, timely treatment, and long-term prevention within the Kingdom's stroke-care framework (100).

National registries and continuous monitoring provide the data needed to evaluate program effectiveness. WSO recommends universal, near-real-time surveillance of stroke incidence, risk-factor prevalence, and treatment adherence (8). Ireland's Stroke Service Tracker and the Saudi Stroke Registry (still under development) exemplify such systems, enabling benchmarking against key performance indicators (17, 102).

Professional training and public-private collaboration address workforce shortages and resource constraints. Task shifting from training Community health workers, nurses, and pharmacists to perform blood pressure checks, lifestyle counselling, and medication titration has been identified as a cost-effective strategy in both high- and low-income settings (95). Public-private partnerships can fund digital platforms, subsidize polypill production, and support community-based wellness programs, thereby amplifying the reach of prevention initiatives (8).

Artificial intelligence (AI) has substantially advanced stroke prevention through the application of machine learning (ML)–based risk prediction models. These approaches also enable the monitoring of post-acute recovery trajectories and facilitate the earlier identification of patients at elevated risk, supporting timely and targeted interventions (108). Algorithms trained on demographic and clinical variables such as age, body mass index, blood pressure, and glucose levels can identify individuals at elevated risk of stroke (109), with data-balancing techniques such as SMOTE (Synthetic Minority Over-sampling Technique), improving model performance on imbalanced datasets (110). In the acute phase, AI is increasingly integral to accelerating diagnosis, imaging interpretation, phenotyping, and clinical decision-making, all of which are crucial for delivering time-sensitive interventions such as thrombolysis and mechanical thrombectomy (111).

### 3.3 Screening and early detection

The primary and most critical step within stroke management is prehospital recognition of the main symptoms of stroke. Most of these symptoms can occur before the patient reaches the hospital for diagnosis, and require public awareness (112). Multiple public health initiatives aim to improve early recognition of stroke and prompt activation of emergency care. Among these, the FAST and BE-FAST tools are widely recommended to aid early stroke identification. FAST emphasizes facial asymmetry, arm drift, speech abnormalities, and the imperative to phone emergency services immediately. BE-FAST enhances FAST by adding Balance (Sudden imbalance) and Eyes (visual disturbance), thereby improving recognition of posterior circulation events. Use of these tools has been reported to identify approximately 70%–90% of strokes and to facilitate timely response, which is critical to outcomes (113–116). In Saudi Arabia, the current landscape of stroke screening and early detection demonstrates meaningful progress but also notable gaps. National initiatives led by the Ministry of Health (MOH) and the Saudi Stroke Society have strengthened public education, particularly through campaigns promoting recognition of stroke symptoms using the FAST and BE-FAST tools. Despite these efforts, several national studies continue to highlight insufficient public awareness. For example, a study conducted in Taif found that only 61.7% of participants could identify stroke-warning signs, underscoring the need for more widespread and effective public health interventions (117).

Following hospital admission, gold-standard methods based on brain and neurovascular imaging are utilized as the first step in stroke diagnosis (118). Among these methods, non-contrast computed tomography (NCCT) is widely used because it is rapid, low-cost, and readily available for brain imaging in patients under evaluation for possible stroke (119). NCCT serves as a highly beneficial tool for examining the penumbra area of the brain and assessing the early clinical phase of ischemic stroke. With this technique, NCCT scans can detect cellular hypoperfusion, cytotoxic edema, and thrombosis that occur during an ischemic stroke (120). NCCT is considered the initial diagnostic imaging modality in most hospital settings and is generally performed to identify the absence of hemorrhagic stroke and intracerebral hemorrhage as the first step in response and treatment (121). Furthermore, NCCT scanning has sufficient sensitivity to detect stroke mimics, such as mass lesions that may arise from tumors, and brain abscesses that may result from infections (122).

Although this method is considered the reference standard for stroke screening, it may have low sensitivity for detecting ischemic stroke in the early hours, as ischemic changes can be subtle. Identifying such changes is challenging and requires a highly experienced neuroradiologist (119). Even with expert-level neurologists or neuroradiologists, the interrater reliability of NCCT diagnosis remains moderate (118). Additionally, NCCT, as a CT-based modality, involves ionizing radiation, which may compromise its safety and limit its use in specific populations, particularly radiosensitive groups (123).

CT Angiography (CTA) is another rapid, non-invasive technique used to assess the intracranial and extracranial vasculature during the acute phase of stroke, offering high sensitivity and specificity (121). Unlike NCCT, which provides only structural brain imaging, CTA distinguishes itself by visualizing cerebral blood vessels via injection of an iodine-based contrast agent, making it especially valuable for detecting vascular abnormalities in patients with suspected stroke (124). Thus, compared with NCCT, the CTA method is more sensitive for detecting large-vessel occlusion (LVO) in stroke patients (125). Multiphase CTA, a specialized form of CTA, offers distinct advantages over conventional CTA in the assessment of acute ischemic stroke. These advantages include improved detection of large vessel occlusions, more accurate evaluation of collateral circulation, greater resilience to patient motion and hemodynamic instability, and higher interrater reliability (126). Similar to NCCT, CTA involves exposure to ionizing radiation. In addition, CTA requires the use of contrast agents, typically iodine-based compounds, which may be unsuitable for patients with allergic reactions or renal impairment (123, 127).

CT Perfusion (CTP), on the other hand, is a CT-based technique that uses dynamic contrast imaging to estimate cerebral blood volume (CBV), cerebral blood flow (CBF), and mean transit time (MTT), thereby allowing identification of the ischemic core and the penumbra (128). Compared to NCCT and CTA, CTP provides information on tissue perfusion status, enabling distinction between the ischemic core and the penumbra, thus supporting more accurate treatment decisions (129). CTP requires additional ionizing radiation beyond NCCT and CTA, and the procedure takes longer compared to NCCT or CTA (118, 128).

MRI, compared to CT-based techniques, provides high sensitivity in acute ischemic stroke during the early phase (112). In addition, MRI offers high resolution for detecting posterior fossa strokes, as it avoids bone-induced artefacts and demonstrates higher sensitivity for small lesions (130). Although MRI provides superior diagnostic capabilities and avoids radiation exposure compared to CT, it remains costly. It is contraindicated in certain patients, such as those with metallic foreign bodies or implantable devices. Also, it is less accessible and is usually adopted for post-treatment imaging assessment (131).

Over recent years, Artificial Intelligence (AI) have shown potential to support stroke imaging interpretation and clinical triage in selected settings. Their clinical value in Saudi Arabia will depend on local validation, workflow integration, and equitable access. AI as technology has incorporated machine learning and advanced algorithms to enhance the interpretation of medical imaging modalities such as CT scans and MRIs (132, 133). For CT, AI-based deep learning has enhanced both image acquisition speed and quality, which enables accurate and rapid identification of stroke. Furthermore, AI has enabled neurologists to interpret low-dose CT images with high fidelity, thereby reducing radiation doses. For CTA/CTP, AI has facilitated the detection of large vessel occlusions (LVOs) and the assessment of collateral circulation with high accuracy, comparable to that of neuroradiologists (134). AI has contributed to automating CT and MRI scans, reducing the time and expertise required for diagnosis (132). AI has created an end-to-end model based on diffusion-weighted imaging (DWI) to identify patients with ischemic stroke who are at risk of AF and to detect hidden AF. In addition, integrating AI with convolutional neural networks (CNNs) has enhanced diagnostic accuracy (135).

At the hospital level in Saudi Arabia, most tertiary centers in major cities are equipped with rapid-access NCCT and CTA, with growing adoption of MRI and CT perfusion within comprehensive stroke centers as part of the evolving national stroke care pathway (136). However, access to advanced neuroimaging remains uneven across regions; many rural hospitals continue to face constraints on imaging availability and limited neuroradiology expertise.

In recent years, Saudi Arabia has undertaken several efforts to enhance early stroke detection, including initiatives under the National Health Transformation Program, expansion of telestroke services, and the establishment of regional stroke hubs (137). Despite these advances, early identification remains the decisive step in improving stroke care. This requires enhanced community awareness and standardized prehospital screening, while emerging tools, such as AI-assisted imaging and rapid, low-cost biomarker platforms, serve further to facilitate timely diagnosis across emergency and remote healthcare settings.

## 4 Therapeutic strategies and clinical practices

### 4.1 Acute stroke care

The goal of therapy in acute stroke is to restore cerebral blood flow to the brain in ischemic stroke and to control bleeding in hemorrhagic stroke (138). In ischemic stroke, achieving vessel recanalization and reperfusion reduces the extent of brain injury. It helps restore neurological function, while in hemorrhagic stroke, hemodynamic stabilization and hematoma control, through medical or surgical interventions, have been shown to limit brain injury and mitigate neurological deterioration (138). Immediate brain and vessel imaging in the emergency department (ED), with the use of NCCT with or without CTA, should be performed to exclude hemorrhagic stroke, and to identify LVO and late window eligibility, guiding appropriate management protocols and reperfusion interventions (55). Patient stabilization including, maintenance of airway function, oxygenation (target SpO₂ ≥ 94%), fever and blood glucose (BG) control (temperature >38 °C and BG 140–180 mg/dL, respectively), blood pressure (BP) control in patients with elevated BP (systolic BP <185 mmHg and diastolic BP < 110 mmHg) should be prioritized before IV thrombolytic therapy (55).

It is emphasized that the acute treatment of ischemic stroke is fundamentally different from the treatment of hemorrhagic stroke. In acute ischemic stroke, treatment is primarily directed toward rapid restoration of cerebral blood flow through intravenous thrombolysis, mechanical thrombectomy when indicated, or a combination of both, together with early antiplatelet therapy and careful regulation of blood pressure and other key physiological variables. In contrast, hemorrhagic stroke management focuses on immediate blood pressure lowering, reversal of anticoagulant or antithrombotic effects when indicated, neurosurgical assessment for hematoma evacuation or cerebrospinal fluid diversion, and vigilant monitoring for elevated intracranial pressure (139). Thrombolytic therapy is not used in hemorrhagic stroke (139).

Although advanced therapies such as intravenous thrombolysis and mechanical thrombectomy are increasingly available in tertiary centers, their national impact remains constrained by delayed hospital presentation, limited public awareness, shortages of stroke-ready facilities in peripheral regions, and variability in prehospital emergency response systems. These findings indicate that improving infrastructure alone is insufficient without parallel investments in public education, emergency transport systems, and equitable access to healthcare.

#### 4.2.1 Intravenous thrombolysis (IVT)

Thrombolysis with intravenous alteplase is primarily associated with improved functional outcomes and reduction of long-term disability after ischemic stroke, while its effect on overall mortality appears modest and not consistently statistically significant across studies (140–143). Alteplase (0.9 mg/kg, max 90 mg over 60 min with the initial 10% of the dose given as an IV bolus over 1 min) within 4.5 h of the patient's last known well (LKW) or stroke symptom recognition remains the recommended thrombolytic of choice per the AHA/ASA guidelines in patients meeting eligibility criteria (55). Recently, the US Food and Drug Administration (FDA) has approved tenecteplase (TNKase) for the management of acute ischemic stroke, administered as a single 0.25 mg/kg IV push within 3 h of symptom onset (144, 145). This approval was based on the results of the intravenous tenecteplase compared with alteplase for acute ischemic stroke in Canada (AcT) non-inferiority trial, which demonstrated that tenecteplase was safe and non-inferior to alteplase in acute ischemic stroke patients who met standard criteria for thrombolysis (146). Stroke management protocols vary across healthcare centers and may now protocolize tenecteplase as an alternative to alteplase, consistent with the European Stroke Organization (ESO) guideline recommendations that favor tenecteplase over alteplase, given its safety, efficacy, and ease of administration (147).

Extended window IVT (4.5 to 24 h) remains debatable in clinical practice. The Thrombolysis in Imaging-Eligible, Late-Window Patients to Assess the Efficacy and Safety of Tenecteplase (TIMELESS) study failed to show the benefit of tenecteplase compared to placebo outside the 4.5-hour window after LKW in LVO, and most patients (77%) proceeded with endovascular thrombectomy (EVT) (148). In the Tenecteplase Reperfusion Therapy in Acute Ischemic Cerebrovascular Events III (TRACE III) trial, patients with LVO in the middle cerebral artery (MCA) or internal carotid artery (ICA) who had salvageable brain tissue identified on perfusion imaging and did not have access to EVT, tenecteplase administered within 4.5–24 h after stroke onset resulted in less disability and similar survival rates compared to standard of care but with numerically higher intracerebral hemorrhage (149). These results support considering perfusion-guided IVT when EVT is unavailable or not anticipated, provided that appropriate imaging is used to select eligible patients (150). In the absence of clear, strong evidence supporting the use of IVT in extended treatment windows, ongoing trials and future pooled meta-analyses of randomized controlled trials (RCTs) will shed more light on its appropriate use (151, 152).

#### 4.2.2 Intra-arterial therapy (IAT)

IAT includes the use of catheter-based administration of thrombolytic drugs, such as alteplase and tenecteplase, directly into or near the occlusion site of the vessel, with or without adjunctive mechanical disruption (153). It provides the benefit of local site clot lysis while minimizing systemic exposure to thrombolytics. Current practice guidelines recommend IAT as a salvage or adjunctive therapeutic option when IV thrombolysis is contraindicated or has failed, or when mechanical thrombectomy is unavailable (154). Early trials of IAT, such as the PROLYSE in Acute Cerebral Thromboembolism II (PROACT II) trial, demonstrated improved recanalization with intra-arterial prourokinase but a higher risk of intracerebral hemorrhage compared with intravenous heparin alone (155). Recent meta-analyses including 6 RCTs showed that IAT after endovascular recanalization improves functional outcomes without increased risk or intracerebral hemorrhage in patients with acute ischemic stroke and LVO, suggesting that IAT may play an essential role in achieving early reperfusion. However, there is no clear consensus on which type and dose of thrombolytic to use in IAT, as studies use heterogeneous agents and doses; therefore, further investigation into the choice of thrombolytic and its doses is warranted (156).

#### 4.2.3 Endovascular thrombectomy (EVT)

EVT in acute ischemic stroke refers to endovascular interventions that achieve recanalization of occluded cerebral arteries using mechanical clot disruption (153). Robust evidence supports the use of EVT for anterior-circulation LVO up to 6 h (157). The AHA/ASA stroke guidelines recommend mechanical thrombectomy in selected patients with acute ischemic stroke and LVO within 6 to 16 h of LKW (55). Moreover, results from the Diffusion-weighted Imaging or CT-perfusion Assessment With Clinical Mismatch in the Triage of Wake Up and Late Presenting Strokes Undergoing Neurointervention With Trevo (DAWN) and the Endovascular Therapy Following Imaging Evaluation for Ischemic Stroke 3 (DEFUSE 3) trials have demonstrated that mechanical thrombectomy can be beneficial in select patients with LVO acute ischemic stroke, even up to 24 h after symptom onset, with clinical-core or perfusion mismatch present (158, 159).

In acute ischemic stroke of the posterior circulation, such as basilar artery occlusion, evidence shows improved outcomes when EVT is used within 6–24 h (160, 161). However, in medium or distal occlusions, recent studies have shown no functional benefit or a neutral effect of EVT compared to usual care (162, 163). Current practice guidelines recommend considering EVT for functionally eloquent M2 occlusions with disabling deficits, after individualized assessment, with the recommendation conditional for M1/ICA and not recommended for more distal branches (M3/M4) (55, 147). EVT is most beneficial when performed within 6 h, and hospitals track performance measures, such as door-to-grain puncture time and from puncture to reperfusion time, to improve outcomes in patients presenting with acute ischemic stroke. However, therapeutic benefits have been shown to persist for up to 24 h, and ongoing clinical trials are investigating whether these effects extend beyond this window (158, 159). Once EVT is performed, BP should be maintained at ≤ 180/105 mm Hg during the procedure and for 24 h after. Furthermore, monitoring blood glucose, fever, and reperfusion injury with brain imaging before starting antiplatelet therapy is recommended (55).

#### 4.2.4 Admission to dedicated stroke units

After IVT or EVT, patients should be admitted to specialized stroke units or neuro-intensive care units. Both AHA/ASA and ESO practice guidelines emphasize that outcomes improve when care is delivered in multidisciplinary stroke units compared to general wards (55, 147). Such high acuity care provides continuous neurological assessment and monitoring, including BP, heart rate, and rhythm, and early recognition of complications, such as hemorrhagic transformation, cerebral edema, and seizures. Additionally, speech and swallow assessments, mobilization, and physical rehab take place in stroke units that consequently increase the chances of recovery following acute ischemic stroke. Integration of these units into hospitals is essential in improving outcomes and reducing death and disability from acute ischemic stroke (164–167).

Despite the growing availability of IVT and EVT in tertiary care centers across Saudi Arabia, the implementation and utilization rates remain suboptimal (35, 61, 168). Local Saudi studies report low rates of alteplase utilization among eligible acute ischemic, primarily due to delayed hospital presentation, limited public awareness of stroke symptoms, prehospital transport delays, and restricted access to specialized stroke centers in peripheral regions (168, 169). Surveys of Saudi neurologists have identified barriers, including limited stroke-ready infrastructure, shortages of trained personnel, delays in neuroimaging, and inconsistent institutional protocols (168). Similar disparities are also evident in secondary stroke prevention, where studies have demonstrated suboptimal adherence to antiplatelet therapy, statins, antihypertensives, and anticoagulation among stroke survivors (170, 171). These findings highlight the need for broader national implementation strategies focused not only on therapeutic availability but also on timely access, adherence, healthcare-system coordination, and long-term follow-up (17).

### 4.2 Secondary stroke prevention

Secondary stroke prevention focuses on determining stroke etiology and comprehensively addressing modifiable vascular risk factors, as underscored by international guidelines, notably AHA/ASA 2021 recommendations (172, 173). In randomized trials and meta-analyses, shared prevention targets encompass blood pressure control to <130/80 mmHg, aggressive lipid reduction with high-intensity statins to attain low-density lipoprotein cholesterol (LDL-C) < 70 mg/dL (or lower for individuals at very high risk), smoking cessation, diabetes mellitus management, weight control, and sustained adherence facilitated by diet, physical activity, and pharmacotherapy. For individuals experiencing transient ischemic attack or non-cardioembolic ischemic stroke, antiplatelet treatment continues to be the fundamental approach for secondary prevention (172, 173).

It is important for long-term therapy to differentiate between secondary prevention after ischemic stroke or hemorrhagic stroke. In ischemic stroke, treatment include high-intensity statin therapy, antiplatelet agents or anticoagulation when indicated, aggressive control of vascular risk factors (hypertension, diabetes, dyslipidemia, smoking), and structured rehabilitation. These strategies aim to reduce the risk of recurrent ischemic events and improve functional outcomes. In patients with a history of hemorrhagic stroke, they may require a different long-term strategy; in particular, statins may be contraindicated or used with caution due to concerns about recurrent intracerebral hemorrhage, and decisions about antithrombotic therapy must be individualized based on hemorrhage type, location, and overall vascular risk profile (174).

Multiple trials have shown that short-term dual antiplatelet therapy works for some high-risk patients, especially those with mild ischemic stroke (NIHSS ≤3) or high-risk transient ischemic attack (ABCD^2^ score ≥4). Research, including INSPIRES, indicates that the combination of aspirin and clopidogrel for 21 to 90 days diminishes the incidence of early recurrent stroke in individuals with large-artery atherosclerosis (172, 173).

Other studies show that taking aspirin and ticagrelor together lowers the risk of ischemic events and death even more than taking aspirin alone. Still, it also raises the risk of significant bleeding. Genotype-guided methodologies have attracted interest, as CYP2C19 loss-of-function alleles correlate with diminished clopidogrel responsiveness; CHANCE-2 demonstrated superior efficacy of ticagrelor relative to clopidogrel in this subgroup (175). Guidelines uniformly recommend maintaining single antiplatelet therapy (aspirin or clopidogrel) after the dual antiplatelet therapy phase (173, 176).

For people who have AF and have had a cardioembolic stroke, anticoagulation is the main way to prevent another stroke. Direct oral anticoagulants are usually preferred over warfarin. Recent trials have elucidated the optimal timing for initiating anticoagulation. The ELAN trial demonstrated that the earlier commencement of direct oral anticoagulants following ischemic stroke was associated with a decrease in ischemic events, without elevating the risk of bleeding (173). Conversely, the TIMING trial illustrated the non-inferiority of early initiation compared to delayed initiation regarding a composite outcome of death, intracranial hemorrhage, or recurrent ischemic stroke (177). On the other hand, anticoagulation is not recommended for embolic stroke of unknown origin without proof of AF. The ARCADIA trial showed that apixaban was not superior to aspirin in patients with embolic stroke of unknown origin with atrial cardiopathy, hence strengthening guidelines to restrict anticoagulation to verified cardioembolic etiologies (173).

Lowering blood pressure is still one of the most reliable ways to prevent subsequent problems. For the majority of patients with post-ischemic stroke or transient ischemic attack, antihypertensive medication is advised once neurological and hemodynamic stability is attained, aiming for a target of <130/80 mmHg. Angiotensin-converting enzyme inhibitors, angiotensin receptor blockers, and thiazide-type diuretics are the most common types of drugs used to treat high blood pressure. Depending on other health problems, they can be used alone or in combination (173). Comprehensive meta-analyses demonstrate that even minor reductions in systolic blood pressure (approximately 5 mmHg) are associated with significant decreases in cardiovascular and cerebrovascular events, particularly in individuals with pre-existing cardiovascular conditions (177).

Evidence also strongly supports lipid control. For secondary prevention, high-intensity statins are preferred. If LDL-C objectives are not met, ezetimibe is advised (173). In individuals at very high risk or intolerant to statins, proprotein convertase subtilisin/kexin type 9 (PCSK9) inhibitors further diminish significant adverse cardiovascular events and ischemic stroke, as evidenced by recent trials and pooled analyses (178, 179). For patients who cannot tolerate statins and when PCSK9 inhibitors are not viable, bempedoic acid serves as an alternate option, with the CLEAR Outcomes trial indicating a decrease in atherosclerotic cardiovascular disease events (175).

Glucose-lowering medications also help lessen the risk of vascular disease in those with cardiometabolic disease. Glucagon-like peptide-1 receptor agonists, such as semaglutide and tirzepatide, reduce the risk of significant cardiovascular events, including stroke, especially when weight reduction or HbA1c reduction is sought. Sodium-glucose cotransporter-2 inhibitors offer substantial advantages for heart failure and renal outcomes; nevertheless, their impact on stroke is predominantly neutral, justifying their application solely for these conditions (177, 178).

Long-term adherence is a critical factor influencing the efficacy of secondary prevention. Recent meta-analyses and trials like SECURE have demonstrated that fixed-dose combination “polypill” techniques can help people stick to their medications and lower the risk of cardiovascular events in secondary prevention populations (177, 178). Team-based follow-up, particularly pharmacist-led medication management, has demonstrated modest yet scalable advantages in enhancing medication adherence (179). Guidelines stress practical interventions like pill organizers, longer prescription refills, help with cost navigation, education in the correct language, and regular reviews of deprescribing to address bleeding risk, falls, drug-drug interactions, and social determinants of health (176).

These evidence-based measures align with Saudi Arabia's national goals under Vision 2030, which focus on preventing non-communicable diseases, expanding specialized stroke facilities, and improving secondary prevention through initiatives led by the MOH. Because recurrent stroke is becoming more common in the Kingdom, combining pharmacologic prevention based on guidelines with system-level interventions like standardized stroke pathways, multidisciplinary follow-up, and digital health solutions is a viable way to cut down on recurrence, disability, and long-term healthcare costs.

### 4.3 Rehabilitation services

Post-stroke rehabilitation constitutes a critical phase in the continuum of stroke care, aiming to restore neurological function, enhance independence in activities of daily living (ADLs), and improve overall quality of life. The rehabilitation process is inherently multidisciplinary, involving physiotherapists, occupational therapists, speech-language pathologists, and increasingly, neurotechnology specialists. The efficacy of rehabilitation depends on early initiation, intensity, and individualized treatment protocols, as emphasized by contemporary guidelines from the AHA and the WHO (180).

Physiotherapy remains the cornerstone of post-stroke motor rehabilitation. It targets impairments in mobility, balance, and coordination through structured exercise regimens and task-specific training. A 2025 network meta-analysis encompassing 41 randomized controlled trials (RCTs) identified dual-task oriented training (DTOT) and constraint-induced movement therapy (CIMT) as the most effective modalities for improving upper limb function and mental health outcomes (181). These interventions leverage neuroplasticity by engaging patients in cognitively demanding motor tasks, thereby enhancing cortical reorganization. In Saudi Arabia, physiotherapy services are expanding yet remain unevenly distributed. A recent study conducted at Sultan Bin Abdulaziz Humanitarian City revealed that while inpatient rehabilitation is available, home-based physiotherapy is limited, particularly in rural regions (182). Barriers include workforce shortages, a lack of standardized protocols, and insufficient integration of community-based services.

Occupational therapy (OT) addresses deficits in self-care, productivity, and leisure activities. It also plays a pivotal role in cognitive rehabilitation and environmental adaptation. The same meta-analysis noted that aquatic and land combined exercise (ALCE) and upper limb training (ULT) significantly improved emotional well-being and communication abilities (181). OT interventions increasingly incorporate virtual reality and robotics to simulate real-world tasks and support feedback-driven learning. Despite its clinical value, OT remains underutilized in Saudi Arabia. The average length of stay (LOS) in rehabilitation centers is 46.71 days, significantly longer than in acute hospital settings (9.07 days), suggesting delayed transitions and potential inefficiencies (183). Moreover, socioeconomic factors such as illiteracy, unemployment, and lack of insurance coverage impede access to OT services (182).

Speech-language therapy (SLT) is essential for managing aphasia, dysarthria, and dysphagia. Emerging techniques such as transcranial alternating current stimulation (tACS) and vagal nerve stimulation (VNS) have demonstrated promising results in enhancing language recovery and motor coordination (184, 185). The Vivistim Paired VNS™ System, for instance, has shown a two- to three-fold improvement in upper limb function when combined with SLT and OT, underscoring the potential of neuromodulation in augmenting traditional therapies (185). In Saudi Arabia, SLT services are constrained by a limited number of certified professionals and inadequate outreach in rural areas. A cross-sectional study found that only 54.9% of stroke survivors had access to a primary care physician, and fewer than 24% possessed disability parking cards, reflecting broader systemic gaps in post-stroke support (182).

The landscape of stroke rehabilitation is rapidly evolving with the integration of non-invasive brain stimulation (NIBS), peripheral stimulation, and brain-computer interfaces (BCIs). Techniques such as repetitive transcranial magnetic stimulation (rTMS), transcutaneous electrical acupoint stimulation (TEAS), and deep brain stimulation (DBS) are being explored for their capacity to modulate neuroplasticity and enhance motor recovery (184, 186). These modalities, while promising, require rigorous validation and substantial infrastructural investment, which currently limits their deployment in Saudi clinical settings.

Geographic, economic, and sociocultural factors influence access to specialized rehabilitation services in Saudi Arabia. Stroke survivors residing outside metropolitan areas face significant barriers, including transportation difficulties, a lack of home modifications, and limited financial aid (182). The healthcare system's reliance on centralized rehabilitation centers exacerbates these disparities, underscoring the need for a shift toward decentralized, community-based models. Furthermore, the economic burden of stroke rehabilitation is substantial. High unemployment rates (73.2%) and lack of medical insurance (84.5%) among stroke survivors underscore the need for policy interventions aimed at improving affordability and accessibility (182). The Vision 2030 initiative offers a strategic framework to address these challenges through investment in digital health, workforce development, and public-private partnerships.

Recent studies have emphasized the effectiveness of telerehabilitation in improving motor function, balance, and ADLs among stroke survivors. An umbrella review of 28 systematic reviews found moderate- to high-quality evidence supporting telerehabilitation as a viable alternative to conventional therapy (187). In Saudi Arabia, mobile phone usage among stroke survivors exceeds 90%, suggesting a strong foundation for implementing remote rehabilitation programs (182). Additionally, virtual reality (VR) and AI-powered robotics are transforming rehabilitation by delivering immersive, personalized therapy solutions (185, 188). These technologies enhance patient engagement and facilitate adaptive learning, particularly in balance and gait training. For instance, recent evidence shows that an AI-assisted hybrid rehabilitation system combining a dual-arm robotic platform with electromyography (EMG)-guided neuromuscular electrical stimulation (NMES) can substantially improve upper-limb motor function. In one study, patients achieved a 44% increase in upper-limb range of motion, demonstrating the potential of intelligent, multisystem rehabilitation platforms to accelerate functional recovery (189).

Community-based rehabilitation (CBR) models are gaining attention as sustainable solutions to address access disparities. A qualitative study in Saudi Arabia highlighted the need for culturally sensitive CBR programs that incorporate education, self-management, and social support (190). Participants emphasized the importance of faith, family involvement, and leisure activities in promoting recovery. Despite these insights, CBR remains underdeveloped, underscoring the need for further research and policy support.

Current rehabilitation services in Saudi Arabia remain disproportionately concentrated in major urban centers, contributing to inequities in long-term stroke recovery. Furthermore, limited integration of community-based rehabilitation and home-based follow-up may reduce adherence and functional outcomes, particularly among elderly patients and individuals living in rural regions.

In conclusion, while Saudi Arabia has made strides in establishing rehabilitation centers and adopting interdisciplinary care models, significant challenges remain. These include geographic inequities, socioeconomic barriers, and limited integration of emerging therapies. Addressing these issues requires a multi-pronged strategy involving policy reform, workforce training, and investment in technology-driven rehabilitation. The Vision 2030 framework provides a unique opportunity to transform stroke recovery services, ensuring equitable access and improved outcomes for all patients.

### 4.4 Trends in clinical practice

The establishment of dedicated stroke units has become a global standard in stroke management, offering structured, time-sensitive care that significantly improves patient outcomes. In Saudi Arabia, several leading hospitals have responded to this paradigm by creating specialized stroke units that follow evidence-based protocols. These units are staffed by multidisciplinary teams including neurologists, emergency physicians, radiologists, nurses, and rehabilitation specialists who work together to manage all phases of stroke care (100, 191, 192).

National policy, such as the standards set by the Saudi CBAHI, has played a key role in promoting these efforts. A national point-prevalence study by Heena and colleagues found that hospitals with stroke units achieved higher compliance with acute stroke guidelines, particularly in the administration of timely thrombolysis and early rehabilitation (76, 191). However, such units are primarily concentrated in urban tertiary centers, leaving rural and peripheral regions with limited access to specialized care.

Historically, stroke treatment was limited by a narrow therapeutic window, particularly the 4.5-hour limit for intravenous thrombolysis. However, breakthroughs in clinical research, such as the DAWN and DEFUSE 3 trials, have shown that some patients with specific imaging profiles can benefit from mechanical thrombectomy up to 24 h after symptom onset (193, 194).

In response, stroke centers in Saudi Arabia are increasingly adopting advanced neuroimaging techniques, such as perfusion CT and diffusion-weighted MRI, to identify candidates for late-window interventions. These changes have been instrumental in expanding treatment eligibility, particularly for patients who experience delayed hospital arrival (195).

Despite these medical advances, logistical challenges remain. Many patients in the Kingdom still arrive outside optimal treatment windows due to low public awareness, limited EMS training, and inefficient transport systems. Addressing these delays through education and improved emergency response infrastructure is crucial for maximizing the benefits of expanded treatment protocols (196).

Telemedicine has emerged as a powerful tool for stroke care, especially in countries with large geographic areas and uneven access to specialists. In Saudi Arabia, the MOH has launched tele-stroke programs that connect remote hospitals with neurologists in larger centers. These systems enable real-time diagnosis and treatment planning, often leading to faster intervention and better outcomes (197).

Although still in early stages, these programs show promise. They are especially beneficial in areas where neurologists and stroke-trained personnel are not readily available. Mobile Stroke Units (MSUs), ambulances equipped with CT imaging and telehealth capabilities, offer an even more advanced solution. Although the Kingdom has not yet widely implemented MSUs, it is considering deploying them in densely populated cities such as Riyadh and Jeddah. Their potential to bring hospital-level care to patients' doorsteps could significantly reduce time-to-treatment and improve survival rates (198, 199).

AI is reshaping how clinicians approach stroke diagnosis and management. AI-powered tools can analyze brain imaging data within seconds, detect large-vessel occlusions, estimate infarct core size, and even predict clinical outcomes. These technologies are proving valuable in emergency departments where time is critical (200, 201). In Saudi Arabia, some hospitals are exploring integrating AI systems into their stroke workflows. These tools assist in triaging patients, selecting appropriate imaging pathways, and determining treatment eligibility. AI is also being tested in post-stroke care, where it helps tailor rehabilitation plans and forecast recovery trajectories (202).

Nevertheless, adopting AI in clinical practice requires careful consideration. Validation studies, staff training, and attention to data privacy are essential before widespread implementation. National stroke registries and data-sharing frameworks would further support AI integration and improve consistency in care delivery.

Saudi Arabia is aligning itself with global trends in stroke care by adopting a more structured, technology-enabled approach. From the growing presence of stroke units and multidisciplinary teams to the use of telemedicine and cutting-edge AI tools, the country is making significant strides to improve patient outcomes. However, challenges such as unequal access to specialized services, delayed treatment, and limited public awareness still hinder progress. Continued investment in infrastructure, education, and innovation will be key to building an equitable, responsive stroke care system that meets the needs of all regions across the Kingdom.

## 5 Innovations and future directions in stroke care

### 5.1 Novel treatment approaches

Stroke therapy is evolving beyond thrombolytics toward multimodal strategies targeting neuroprotection, immunomodulation, bridging therapies, and neurorepair, supported by innovations in drug delivery, AI, and regenerative medicine. Pharmacological stroke therapy has evolved significantly, moving beyond conventional thrombolytics like intravenous tissue plasminogen activator (tPA) toward innovative strategies aimed at enhancing efficacy, mitigating secondary brain injury, and promoting long-term neurorepair. Novel drugs, such as caADAMTS13, are being developed to target von Willebrand factor (vWF) to prevent abnormal platelet aggregation and microvascular thrombosis. In animal models, caADAMTS13 has been shown to reduce lesion volume and minimal neurological deficits compared to tPA (203). Another area of innovation is targeting excitotoxicity with neuroprotective agents like nerinetide, which a meta-analysis of three trials found to have its maximum effect when administered within three hours of stroke onset. This finding aligns with preclinical data (204, 205). Furthermore, innovations in nanoparticle-based delivery systems are helping drugs to cross the blood-brain barrier, increasing their specificity and efficacy, and offering the potential for more personalized stroke therapies (206).

Cell and gene therapy are promising strategies for improving stroke outcomes by promoting neuroprotection, angiogenesis, and neuroregeneration. The progress of cell-based therapies for stroke has reached the translational stage, with over 500 clinical trials initiated by the end of 2023 to investigate their benefits for patients (207). In these trials, mesenchymal stem cells (MSCs) are the predominant cell type due to their accessibility and immunomodulatory properties. While these therapies have achieved favorable safety and tolerance profiles, their efficacy remains mixed and inconclusive. This is mainly due to the heterogeneity across studies—including variations in MSC sources, trial designs, patient selection, and dosage—as well as small sample sizes (207). Future research is expected to increasingly focus on alternative cell types, such as neural stem cells and induced pluripotent stem cells (iPSCs), which have the unique potential to directly replace damaged or lost neurons and contribute to the functional repair of the injured brain (208, 209).

On the other hand, gene therapy in stroke focuses on delivering genetic material, such as DNA, RNA, or siRNA, to specific brain cells to target the molecular mechanisms of brain injury and repair. For instance, the delivery of a vascular endothelial growth factor (VEGF) gene shows promise in stroke therapy by enhancing the formation of new blood vessels and improving blood flow to the ischemic area. A preclinical study using a rat model of ischemic stroke demonstrated that a single administration of an adeno-associated virus (AAV) expressing human VEGF (AAV-VEGF), given one day post-ischemia, promoted functional angiogenesis and neurogenesis when examined eight weeks later (210). Additionally, targeting upregulated genes post-stroke, such as Tenascin-C and *α*-Synuclein, with siRNA can mitigate neuroinflammation and secondary brain damage, thereby enhancing neurological recovery (211, 212).

Recent studies have highlighted that stroke is not solely a vascular event but is also characterized by a pronounced immune and inflammatory response, which plays a critical role in determining the severity of neuronal damage and influencing long-term functional outcomes. Within this framework, immunotherapeutic strategies hold promise for improving stroke prognosis by mitigating secondary brain injury and enhancing mechanisms of neural repair and recovery (213). In a preclinical study, the administration of a small molecule (AX-024) successfully inhibited the antigen-specific T-cell response within 24 h of intracerebral hemorrhage in a mouse model, leading to improved neuroinflammation markers compared with the control group (214). Meanwhile, an IL-1 receptor antagonist was evaluated in a Phase II clinical trial on ischemic stroke patients. When administered subcutaneously within five hours of onset, the results showed a reduction in inflammatory markers, including IL-6 and CRP (215). However, despite this effect, there was no significant improvement in the patients' clinical outcomes, highlighting the need for larger, adequately powered clinical studies to determine if the anti-inflammatory effect translates into a meaningful clinical benefit.

Stroke-induced brain injury can lead to significant alterations in the gut microbiota, a condition known as dysbiosis. This dysbiosis, in turn, can exacerbate systemic inflammation and neuroinflammation, thereby hindering recovery. Therefore, targeting the microbiome-gut-brain axis has emerged as a novel approach to improve stroke outcomes. Probiotics have demonstrated several benefits in stroke management, primarily by reducing complications, improving nutritional status, and enhancing recovery. In a randomized clinical trial of 120 patients admitted to the ICU after a stroke, participants received probiotic supplementation. The 60 patients who received probiotics showed improved levels of inflammatory markers (IL-10 and IL-6) and reduced oxidative stress, as well as a lower incidence of pathogenic infections (216). In addition, fecal microbiota transplantation (FMT) has been shown in preclinical studies to help reverse the gut microbiota changes caused by the stroke and attenuate ferroptosis, as demonstrated in a preclinical study (217).

Several non-invasive neuromodulatory strategies have emerged as promising additions to conventional stroke rehabilitation, supported by both preclinical and clinical evidence. Repetitive Transcranial Magnetic Stimulation (rTMS) has been shown to modulate cortical excitability and enhance neuroplasticity in animal models (218). A recent meta-analysis of 70 studies involving 2,951 stroke survivors suggested that applying low-frequency rTMS (1 Hz) over the primary motor cortex can enhance motor function (219). Similarly, transcranial direct current stimulation (tDCS) has shown promising results in enhancing post-stroke cognitive and motor skills (220). A major area of innovation in this field is the combination of these non-invasive techniques with other rehabilitation methods to create more powerful, synergistic therapies.

### 5.2 Personalized and AI-driven medicine

Innovation and future trends in stroke care are increasingly characterized by the integration of personalized medicine, AI, advanced genomics, and novel pharmaceutical technologies, including 3D printed medication, resulting in a paradigm shift in prevention and treatment strategies (221, 222). Stroke, a primary cause of morbidity and mortality globally, poses significant challenges due to its diverse etiology, varying clinical manifestations, and limited therapeutic windows of existing pharmacological treatments and reperfusion methods (223). Conventional care models have implemented generalized stroke preventive measures that highlight well-recognized vascular risk factors, including hypertension, diabetes, and hyperlipidemia, and nevertheless, they fail to include the interindividual variability in stroke causation and treatment efficacy (223). In contrast, precision medicine, powered by genomics and AI-driven tools, is emerging as a game-changing approach to address the unmet need for individualized risk profiling and tailored therapeutic interventions (221, 224).

Genomic technologies provide critical insights into polygenic risk scores and rare genetic variants that predispose patients to ischemic or hemorrhagic subtypes, enabling earlier identification of individuals at elevated risk and guiding stratification-based prevention strategies (224). For example, genome-wide association studies (GWAS) are revealing significant risk loci linked to AF or dyslipidemia and, when coupled with polygenic algorithms, can accurately estimate a patient's likelihood of future stroke events more accurately than a conventional epidemiological model (224). Moreover, pharmacogenomic research has emphasized the importance of genetic variants influencing drug metabolism and anticoagulant responses, such as cytochrome P450 and VKORC1 polymorphisms for warfarin, and variability in clopidogrel bioactivation pathways, highlighting the need for individualized prescribing regimens to optimize both efficacy and safety (225). The high prevalence of hypertension, diabetes, obesity, and smoking reported across Saudi studies strongly supports prioritizing preventive strategies and population-level risk reduction policies.

AI is a transforming element in stroke care, facilitating data-driven clinical decision making in prevention, acute management, and rehabilitation (226). Through the integration of electronic health records, imaging, genomics, and real-time monitoring via wearable devices, AI algorithms may produce sophisticated individualized risk profiles that adjust according to continuous lifestyle, biomarker, or cognitive alterations (223, 227). In acute stroke, prompt triage and timely reperfusion are essential. AI-assisted imaging platforms have proven superior in identifying subtle ischemic lesions and quantifying perfusion deficits beyond the capabilities of radiologist interpretation alone, thereby expediting door-to-needle times and enhancing thrombectomy candidacy selection (221, 223). In addition to acute imaging, AI-driven predictive models are being investigated for long-term secondary prevention, as they can use genomic data, comorbidities, and drug adherence habits to predict recurrent stroke risks and recommend personalized preventive strategies (223, 226). Moreover, deep learning methods that incorporate wearable data, including blood pressure variability, cardiac rhythm monitoring, and glucose changes, offer proactive early warnings of cerebrovascular instability before the onset of symptomatic episodes (227).

The integration of genomic and AI-driven personalization with the emerging field of 3D-printed pharmaceuticals promises a future in which stroke patients receive treatments specifically matched to their genetic and metabolic profiles and individual dosing needs (228, 229). Stroke patients frequently require a complex regimen of antiplatelets, antihypertensives, lipid-lowering, and anticoagulants, rendering adherence a significant struggle. 3D printing can resolve this issue by manufacturing fixed-dose combination “polypills” that integrate several drugs into a single, personalized tablet, engineered with precise pharmacokinetic and release profiles tailored to an individual's physiology or chronotherapy requirements (230). Printable formulations can address dysphagia, which is a prevalent issue in stroke patients, by producing readily dissolvable or chewable dosage forms, hence enhancing patient compliance and recovery (231). In addition to traditional pharmaceuticals, 3D printing offers promise in innovative neurorestorative methods, such as delivery matrices for stem cells and neuroprotective peptides tailored for individualized regeneration strategies (232).

These improvements indicate a thorough rethinking of stroke prevention and care, customized for both patient demographics and individual needs (233). Genomics enables the identification of individuals at risk and the characterization of their biological responses to pharmaceuticals. At the same time, AI platforms analyze multifactorial data to provide predictive insights and tailored algorithms, and 3D printing converts these insights into practical therapies optimized for compliance, safety, and recovery. Challenges persist in ethical data management, algorithmic transparency, equality, and regulatory advancement for 3D-printed pharmaceuticals (228). The clinical trajectory suggests a future in which stroke care becomes more proactive, personalized, and precision-oriented, enhancing efficiency in acute therapies and significantly improving preventive and rehabilitation outcomes (233). Utilizing genetics, AI, and 3D printing within a cohesive framework, stroke therapy is poised to evolve from reactive treatment to personalized prediction and prevention, ultimately converting stroke from an unpredictable emergency into a treatable, individualized illness (234).

Saudi Arabia is increasingly incorporating precision-medicine frameworks and AI-enabled technologies into its national stroke-care pathways. The country's ongoing Saudi Human Genome program and the digital health transformation have created a foundation for standardized, data-driven acute stroke assessment—particularly valuable for hospitals that lack on-site stroke specialists. Precision-medicine initiatives aligned with the national research priorities set by the Saudi NIH, MOH, and the Research, Development, and Innovation Authority (RDIA) are advancing biomarker-based risk stratification, including genomic profiling, cardioembolic risk prediction tools, and blood-based biomarkers that differentiate ischemic from hemorrhagic stroke or assess tissue viability.

Despite this progress, several challenges remain, including regional disparities in access to advanced neuroimaging, the limited availability of large, annotated clinical datasets needed for robust AI model development, and the ongoing need to clinically validate predictive algorithms within the Saudi population to ensure accuracy and generalizability. While AI-based diagnostic systems show promising accuracy in stroke imaging interpretation, implementation in Saudi Arabia remains limited by infrastructure variability, data integration challenges, and the need for local validation studies.

### 5.3 Healthcare policy and system-level improvements

The MOH has introduced the National Stroke Readiness Standards to streamline patient pathways from symptom identification to rehabilitation across all hospitals and ensure consistent access to evidence-based acute treatments. The standards are intended to reduce the burden of stroke and transient ischemic attack by lowering mortality and morbidity, enhancing patient experience, improving accessibility, and directly supporting Saudi Vision 2030 by improving population health, enhancing care experiences, and reducing per capita spending (235). Moreover, the MOH created an accreditation pathway for stroke centers across the country based on previously mentioned standards and aligned their protocols with internationally recognized guidelines, such as the Australian Clinical Guidelines for Stroke Management. The overall goal of this movement is to ensure rapid triage, timely imaging, and standardized access to reperfusion therapies (e.g., thrombolysis and thrombectomy) (137, 235).

The Council of Health Insurance (CHI) in Saudi Arabia has released a document outlining several quality markers for good service. Each quality marker is mapped to multiple criteria that aim to improve the quality of service across the stroke pathway. In addition, several key performance indicators (KPIs) are associated with the standards, including the median time intervals from door to CAT scan and from door to needle. Collectively, these markers set an agenda to deliver the best possible stroke services, from prevention to lifelong support (100).

At the system level, Saudi Arabia is working to expand stroke-specific infrastructure and workforce by dedicating stroke units within the newly formed health cluster networks managed by the Saudi Health Holding Company, as only about 5% of stroke patients were previously admitted to specialized units (236). The MOH has prioritized investment to increase the number and geographic spread of stroke centers, ensuring equitable access across urban and rural regions. Multiple digital health platforms support these operational expansions—most notably the Seha Virtual Hospital, which provides telestroke consultations to rural hospitals, and the 937 Call Center, which facilitates early identification and urgent referrals, thereby streamlining the entire chain of care (237).

Workforce planning studies anticipate a need for a high number of additional specialized professionals, such as stroke neurologists, interventional neuro-radiologists, and occupational and speech therapists, to close the gap between current capacity and international standards. The anticipated rise in demand and the planned model of care will require a substantial expansion of stroke specialists across several disciplines. Specifically, projections indicate a need for 43.8 FTE (full-time equivalent) stroke neurologists and 53.5 FTE interventional neuro-radiologists to provide the necessary expertise to manage stroke patients through 2029 (76). Modeling of ischemic stroke care has quantified the clinical and economic benefits of expanded stroke services—projecting $602 million in savings over 15 years and significant gains in functional independence (18). Moreover, the private sector is contributing to ensuring early access to stroke management, complementing current public initiatives and designating zone-based stroke hospitals. The partnership with the private sector aims to expand its telestroke network through the Seha Virtual Hospital, scaling from 11 hospitals to national coverage. This collaboration includes training healthcare providers, launching public awareness campaigns on stroke symptoms and risk factors, and building a national stroke registry to guide data-driven decision-making and quality improvement across the system (238, 239).

Enhancing stroke prevention in Saudi Arabia requires a more systematic national approach that integrates primordial prevention, primary prevention, and early detection of at-risk individuals. Screening efforts should target individuals with major modifiable risk factors, including hypertension, diabetes mellitus, dyslipidemia, obesity, smoking, physical inactivity, atrial fibrillation, and established cardiovascular disease (17). Additionally, increased public awareness campaigns, behavioral intervention strategies, and routine primary healthcare screening for risk factors would facilitate earlier detection, treatment, and a decrease in future stroke incidence. Meanwhile, the Saudi National Stroke Readiness Standards offer a critical strategy in standardized stroke-care pathways, extending access to evidence-based services throughout health facilities (240). Nonetheless, prevention and monitoring of risk factors remain critical elements to be further integrated into overall healthcare and reinforced under health transformation initiatives.

Healthcare accessibility remains a key factor in optimizing the effectiveness of stroke prevention and management. In Saudi Arabia, citizens have access to free, state-funded care through public hospitals and clinics, whereas private-sector healthcare is regulated through the health insurance system (241). Although this reduces many financial barriers to preventive and secondary stroke care for public healthcare users, other barriers may still impede service uptake. These include waiting times, referral delays, physician availability, regional disparities in service distribution, continuity of follow-up, and variable insurance coverage among private healthcare users. These factors may affect access to screening, optimal risk-factor management, pharmacological preventive treatments, rehabilitation, and long-term secondary prevention (242). Therefore, improving equitable access to prevention services, medications, and ongoing care across different population groups remains essential for reducing stroke burden. Collectively, these efforts, ranging from national standards and telestroke networks to workforce development, primary prevention, and community-based risk-factor control, are transforming Saudi Arabia's approach to stroke care, aiming for more equitable access, better outcomes, and long-term sustainability across the Kingdom (17).

## 6 Study limitation and future perspectives

This review should be interpreted in light of several important limitations. First, much of the available evidence on stroke in Saudi Arabia is derived from single-center or regional studies, which may limit its generalizability to the national level. Second, the lack of comprehensive stroke registries limits the availability of standardized epidemiological and outcome data. Third, as this review was intended to provide a broad narrative overview, some specialized topics could not be addressed in exhaustive detail. Nevertheless, the review offers an updated synthesis of current evidence and highlights major gaps, challenges, and future opportunities in stroke care in Saudi Arabia.

Table 2 summarizes the key challenges, evidence gaps, and future priorities related to stroke prevention, diagnosis, treatment, rehabilitation, and surveillance, while emphasizing the gap between the increasing stroke burden and current limitations in healthcare infrastructure, public awareness, and long-term monitoring systems.

**Table 2:** Key challenges, evidence gaps, and future priorities in stroke care in Saudi Arabia.

Domain	Current Challenges in Saudi Arabia	Evidence/Observations from Literature	Future Priorities
Prevention	High prevalence of hypertension, diabetes, obesity, and smoking	Modifiable risk factors account for most stroke burden	Prevention campaigns and risk-factor screening
Public Awareness	Low recognition of stroke symptoms	Delayed hospital presentation common	FAST/BE-FAST education campaigns
Acute Stroke Care	Unequal access to stroke centers and EVT	Services concentrated in tertiary urban hospitals	Expansion of stroke-ready centers and telestroke
Emergency Response	Delays in EMS activation and transport	Many patients arrive outside the thrombolysis window	Strengthening EMS stroke pathways
Rehabilitation	Limited rehabilitation access in rural regions	Long LOS and insufficient community rehab	Telerehabilitation and decentralized rehab
Surveillance	Lack of a unified national registry	Most studies are regional/single-center	Sustainable stroke registry
Research & Innovation	Limited local AI validation studies	AI adoption remains early-stage	Saudi-specific AI and precision medicine research

## 7 Conclusion and future directions

Stroke remains a significant public-health challenge in Saudi Arabia, with available evidence highlighting a substantial disease burden and persistent gaps in prevention, early diagnosis, timely acute treatment, equitable access to specialized services, and long-term rehabilitation. Although advances in hospital care and emerging therapeutic modalities offer opportunities to improve outcomes, the widespread prevalence of modifiable risk factors underscores the importance of comprehensive prevention strategies. These should include community awareness campaigns, early screening of individuals at risk, lifestyle interventions, and strengthened public-health policies to reduce vascular risk factors and prevent first and recurrent strokes.

Improving stroke outcomes will require strengthening emergency medical services, expanding access to specialized stroke centers, and standardizing evidence-based protocols for acute treatment and secondary prevention. Equally important are investments in rehabilitation infrastructure and integrated care pathways that ensure continuity from prevention and emergency management to rehabilitation and long-term follow-up. Such measures are essential to reducing stroke-related disability, minimizing health inequalities, and improving quality of life. A key priority is to strengthen stroke surveillance through sustainable national registries, standardized data collection, and representative population-based research. Because much of the available Saudi evidence derives from regional and hospital-based studies, the national burden and temporal trends cannot yet be characterized with complete precision. More comprehensive epidemiological data would enable reliable estimation of stroke incidence and outcomes, identification of regional disparities, assessment of healthcare needs, and evidence-informed resource allocation and policymaking. Regular population-based surveys of stroke risk factors would further support the development and evaluation of targeted prevention strategies.

Emerging innovations, including AI-assisted diagnosis, precision medicine, telemedicine, and technology-enabled rehabilitation, offer promising opportunities to improve stroke detection, treatment decisions, and recovery. However, their implementation in Saudi Arabia should be guided by local validation, clinical feasibility, cost-effectiveness, and rigorous evaluation of patient outcomes. These priorities align with Saudi Arabia's Vision 2030 objectives for developing a more efficient, sustainable, and equitable healthcare system. Ultimately, combining stronger epidemiological evidence with sustained investment in prevention, established stroke-care pathways, equitable access to specialized services, and innovative technologies will be essential to reducing the burden of stroke and improving outcomes across the Kingdom.
